# Successful treatment of gastric cancer 10 years after heart transplantation: A case report

**DOI:** 10.1097/MD.0000000000037841

**Published:** 2024-04-19

**Authors:** Lin Zhao, Ye Wang, Zhenggang Guo

**Affiliations:** a Department of Anesthesiology, Peking University, Shougang Hospital, Shijingshan District, Beijing, China.

**Keywords:** case report, gastric cancer, heart transplantation, MDT

## Abstract

**Background:**

While survival rates among cardiac allograft recipients have improved, there has been a rise in post-transplant malignancies, with gastric cancer being less commonly reported. This study presented a successful treatment of gastric cancer in an individual 10 years after undergoing a heart transplant.

**Case presentation:**

A 66-year-old Chinese man presented to the gastrointestinal clinic with a complaint of diagnosis of gastric cancer for 4 months and treated with neoadjuvant therapy for 1 month. He has undergone orthotopic heart transplantation 10 years earlier due to a myocardial infarction. Physical examination and laboratory tests did not reveal any significant abnormalities. Abdominal contrast-enhanced computed tomography (CT) imaging indicated a gastric mass near the greater curvature, with gastroscopy suggesting a carcinoma at the esophagogastric junction, Siewert III. An echocardiogram indicated left atrial enlargement with mild mitral and tricuspid regurgitation. The diagnosis suggested that his gastric cancer at the esophagogastric junction was a consequence of long-term immunosuppressive therapy. A multidisciplinary team (MDT) consultation recommended a proximal radical gastrectomy. Postoperatively, the patient received 4 cycles of adjuvant chemotherapy with XELOX combined with Herceptin, initiated a month after surgery. During the 1-year follow-up, the patient showed commendable recovery, with no signs of tumor recurrence or metastasis.

**Conclusion:**

This case underscores the potential risk of malignancy from immunosuppressive agents in transplant recipients. The successful management of this complex scenario underscores the indispensable role of an MDT approach in treating such unique and challenging cases.

## 1. Introduction

The long-term survival of cardiac allograft recipients has markedly improved due to post-surgery immunosuppressive therapies.^[1]^ Nevertheless, this progress is shadowed by an increased incidence of malignancies. Notably, the first-year cumulative incidence of malignancies stands at 2.9%, surging to 31.9% by the tenth years post-transplantation.^[2]^ Several studies have pointed out a disproportionately higher occurrence of gastric cancer in organ transplant patients compared to the general populace, which is concomitant with higher cancer-related mortality rates.^[2–5]^ Compounding the challenge, cardiac allograft recipients often manifest varying extents of cardiac allograft vasculopathy, introducing significant complexities into perioperative anesthesia management.^[6–8]^ Consequently, treating tumors in post-cardiac allograft patients requires a nuanced approach. The multidisciplinary team (MDT) approach has become pivotal in navigating these intricate cases, aiming to improve patient outcomes. This study elucidates the case of a cardiac allograft recipient diagnosed with gastric cancer, who, after undergoing preoperative chemotherapy, successfully received a proximal radical gastrectomy.

## 2. Case presentation

### 2.1. Chief complaints and history of present illness

A 66-year-old Chinese man presented to the gastrointestinal clinic with a complaint of diagnosis of gastric cancer for 4 months and treated with neoadjuvant therapy for 1 month.

### 2.2. History of past illness

He underwent orthotopic heart transplantation in 2011, following a myocardial infarction. Post-transplant, he had been on anti-rejection medication, including oral prednisone acetate, tacrolimus, mycophenolate mofetil, and trimethoprim tablets. He had been diagnosed with gastric cancer (Siewert III) 4 months ago and had undergone neoadjuvant therapy in the past month.

### 2.3. Personal and family history

His 2 brothers died of pancreatic cancer and esophageal cancer, respectively.

### 2.4. Physical and laboratory examination

Physical examination and laboratory tests did not reveal any significant abnormalities.

### 2.5. Imaging examinations

A contrast-enhanced abdominal CT scan displayed a thickened gastric wall and a pronounced circular mass near the greater curvature. Gastroscopy identified a carcinoma at the esophagogastric junction, classified as Siewert III. An echocardiogram revealed an enlarged left atrium along with mild mitral and tricuspid regurgitation.

### 2.6. Final diagnosis

He was subsequently diagnosed with gastric cancer at the esophagogastric junction, likely a consequence of prolonged immunosuppressive therapy following his heart transplant.

### 2.7. Treatment

The MDT, comprising physicians from Gastroenterology, Cardiology, Cardiothoracic Surgery, Anesthesiology, and Oncology, recommended a proximal radical gastrectomy. A 500 mL glucose-sodium chloride solution was administered intravenously before surgery. Upon entering the operating room, vital signs were stable, and a radial artery puncture was performed for monitoring. Anesthesia was induced with 30 μg sufentanil, 20 mg etomidate, and 20 mg cisatracurium, followed by tracheal intubation. During the right internal jugular vein puncture, the patient blood pressure dropped but stabilized with rapid fluid administration and norepinephrine. Before surgery, 5 mg oxycodone was given for pain relief and sevoflurane-maintained anesthesia.

The abdominal assessment showed no metastasis, and radical resection of distal gastric cancer was completed. Pathological findings revealed a moderately differentiated adenocarcinoma with focal extracellular mucus and extensive inflammatory reactions, graded as TRG 2. The tumor was staged as ypT3N2. Immunosuppressive drugs were administered on the first postoperative day. Their blood concentrations were measured every 3 days, and the dosage was adjusted accordingly. The patient was discharged from the hospital on the 10th postoperative day. Four cycles of XELOX + Herceptin adjuvant chemotherapy were started 1 month after surgery.

### 2.8. Outcome and follow-up

During the 1-year follow-up, no signs of tumor recurrence or metastasis were found. Nevertheless, the patient continued to have difficulty eating, largely limiting his intake to semi-runny foods.

## 3. Discussion

This case report highlights the potential malignancy risk associated with the use of immunosuppressive drugs in heart transplant recipients. The treatment of this intricate situation further emphasizes the vital role of MDT in addressing such distinct and complex cases.

Immunosuppression significantly contributes to post-transplant malignancies. It has been found that cardiac recipients, due to intensive immunosuppressive therapy, had a heightened risk of cancer deaths primarily from lung cancer.^[3]^ Patients over 40 years old face a higher risk of developing tumors post-transplantation.^[9]^ Furthermore, cancers in transplant recipients tend to be biologically more aggressive. As a result, their survival rate is reduced compared to cancer patients in the general population.^[10]^ It is imperative to prioritize regular cancer screenings for long-surviving post-transplant patients. However, gastric cancer following heart transplantation has been less reported. In this study, we reported a case with gastric cancer following heart transplantation a decade later. In addition, the tumor was classified as ypT3N2 and was successfully removed through radical resection, suggesting a potentially favorable prognosis.

Managing patients diagnosed with tumors after organ transplantation is complex due to limited data on treatment efficacy and potential toxicities in this population. The effects of immunosuppressive drugs on various organs, compounded by other health issues, further complicate the matter. Oncologists are hesitant about administering standard chemotherapy or radiation to these patients, fearing severe side effects given their immunosuppressed state. Consequently, transplant recipients with cancer are less likely to undergo surgery and radiation.^[11,12]^ It has been suggested that the dose of chemotherapy drugs needed to be reduced in the initial stage of post-transplantation patients, and only 12% received the full planned standard chemotherapy regimen.^[13]^ In the present study, an MDT discussion could provide individualized recommendations for patient care. The tumor was successfully resected and the patient did not experience serious side effects during the following chemotherapy and targeted therapy.

During the perioperative period, it is essential to maintain stable levels of immunosuppressants to avoid acute rejection. Postoperative assessment of liver function and immunosuppressant levels is vital. Early administration of immunosuppressants and enteral nutrition via a nasogastric tube is feasible. Presently, there a lack of robust evidence supporting specific perioperative use of immunosuppressive drugs.^[14]^ Consistent with other studies, this patient continued immunosuppressive treatment on the day of the surgery and resumed tube feeding on the first postoperative day.

In conclusion, this study highlights the potential risk of gastric cancer from immunosuppressive agents in heart transplant recipients. The handling of this intricate situation suggests the vital importance of an MDT approach for addressing such distinct and challenging cases.

## Author contributions

**Conceptualization:** Lin Zhao, Zhenggang Guo.

**Formal analysis:** Lin Zhao, Ye Wang.

**Investigation:** Lin Zhao, Ye Wang.

**Methodology:** Lin Zhao.

**Resources:** Lin Zhao, Ye Wang.

**Software:** Lin Zhao, Ye Wang.

**Validation:** Lin Zhao, Ye Wang, Zhenggang Guo.

**Visualization:** Lin Zhao, Zhenggang Guo.

**Writing – original draft:** Lin Zhao, Ye Wang, Zhenggang Guo.

**Writing – review & editing:** Lin Zhao, Zhenggang Guo.
